# Investigating the effect of zinc supplementation on probability of relapse and mental health in patients with opioid use disorder undergoing methadone maintenance treatment

**DOI:** 10.1186/s13011-023-00514-5

**Published:** 2023-01-06

**Authors:** Zahra Amini, Ebrahim HeidariFarsani

**Affiliations:** 1 Department of Community and Family Medicine, School of Medicine, Isfahan University of Medical Sciences, Hezar Jerib Street, Isfahan, 8174673461 Isfahan Province Iran; 2 Department of Community Medicine, Isfahan University of Medical Sciences, Isfahan, Iran

**Keywords:** Methadone maintenance treatment, Zinc supplementation, Probability of relapse, Mental health

## Abstract

**Background:**

Considering different factors, such as high withdrawal rates in methadone maintenance treatment (MMT) programs alongside mental health (MH) problems appearing in patients with opioid use disorder and the lack of prior research on the effect of zinc supplementation in this respect, the present study aimed to investigate the effect of zinc supplementation on the probability of relapse (PoR) and MH problems in patients with opioid use disorder undergoing MMT.

**Methods:**

For this purpose, a randomized controlled trial with a clinical basis was fulfilled on a total of 68 patients with opioid use disorder receiving MMT, allocated to two groups, viz. intervention, and control (each one consisting of 34 individuals). Then, the participants in the intervention group were given zinc supplements combined with methadone for three months, and the controls only took methadone, according to the treatment plan. The data were collected using the Relapse Prediction Scale (RPS) and the Depression, Anxiety, and Stress Scale 21 (DASS-21) before, one month after, and at the end of the intervention program.

**Findings:**

Compared to the control group, the likelihood of drug use (*p* = 0.01), drug craving (*p* = 0.002), and the RPS total score (*p* = 0.002) in the intervention group was significantly lower. Moreover, the results revealed a significant decreasing trend in depression (*p* = 0.01), anxiety (*p* < 0.001), stress (*p* = 0.001), and the DASS-21 total score (*p* = 0.001) in the intervention. Compared to the control group, the DASS-21 total score (*p* < 0.001) in the intervention group was significantly lower.

**Conclusion:**

Accordingly, it was concluded that zinc supplementation could reduce the PoR and improve MH problems in patients with opioid use disorder experiencing MMT. However, further research is recommended to fill the gaps.

**Trial registration:**

The research protocol has also been listed on the Iranian Registry of Clinical Trials (IRCT) with code no. IRCT2020050904736N1.

## Background

As one of the first-line treatments for opioid use disorder, methadone maintenance treatment (MMT) seems to be a long-acting or permanent therapy that replaces opioids with other safe alternatives to control physical and mental problems facing patients with opioid use disorder [1]. This type of treatment is being implemented in numerous countries for patients with opioid use disorder and was particularly put into operation in 60 out of 70 countries as opioid treatment providers in 2009 [2]. Although MMT is a form of physical addiction, it is not roughly equivalent to addiction because here, individuals can divest themselves of euphoria, dependence, and forced use of drugs with regular medication intake. MMT also helps patients with opioid use disorder return to the community and concentrate on other aspects of life [3].

The evidence further suggests that many patients with opioid use disorder undergoing MMT suffer from mental health (MH) problems, including depression and anxiety [4, 5]. In this line, one study revealed that 18% of patients with opioid use disorder subjected to MMT had put up with mild to very severe anxiety, 2.8% of them had shown some symptoms of depression, and 4% of the subjects had experienced stress [1]. Accordingly, MH problems could be thought of as negative driving forces, reducing MMT consequences, such as engaging in high-risk HIV-related behaviors that might interfere with MMT adherence and increase methadone tolerance [6]. Identifying such problems and taking appropriate measures are thus essential to multiply the effect of MMT programs for patients with opioid use disorder undergoing MMT.

A noteworthy achievement in such programs is sustainability. Higher relapse rates in this respect mean that a large number of patients with opioid use disorder discontinue treatment and start drug use; therefore, therapy is not effective [7]. Many researchers have further reported that only 26–72% of patients with opioid use disorder adhere to MMT over one year and stand in front of relapses [8, 9]. This has been approximately 30–50% among patients with opioid use disorder in Iran [10, 11], calling for appropriate measures and strategies to boost adherence to MMT programs.

Of note, a healthy, balanced diet containing essential micronutrients supports effective treatment for patients with opioid use disorder undergoing MMT [12]. Following iron, zinc is the second micronutrient, most abundant in the human body, with major catalytic and structural roles. This nutrient can significantly contribute to regulating gene expression in the body, the central nervous system (CNS), the postsynaptic activities, such as hormone secretion and nerve impulse transmission, as well as the postsynaptic proteins [13], and can even strengthen the immune system and help the brain to function properly [14]. It has commonly been assumed that receiving the recommended daily amount of zinc can demote dependence on opioids, which has been supported by the low levels of this nutrient in patients with opioid use disorder [15]. One study had accordingly demonstrated that taking micronutrients, including zinc, had been low before the onset of MMT and even five years after its completion [12].

Behavioral impairments, depression, affective dysregulation, anxiety, aggression, and irritability, as the disorders that are predominantly associated with CNS dysfunction, often occur due to zinc deficiency, as evidenced in human and animal specimens [16–18].

## Aim

Considering the high withdrawal rate in methadone maintenance treatment (MMT) programs alongside mental health (MH) problems appearing in patients with opioid use disorder and the lack of prior research on the effect of zinc supplementation on PoR and MH status in patients with opioid use disorder undergoing MMT, the present study aimed at:

- Investigating the effect of zinc supplementation on PoR in patients with opioid use disorder undergoing MMT
- Investigating the effect of zinc supplementation on MH status in patients with opioid use disorder undergoing MMT

## Methodology

### Research design

This study was a randomized, single-blind, parallel, two-arm clinical trial on patients with opioid use disorder receiving MMT in an addiction treatment clinic in an urban area of ​​Iran from April 2021, lasting three months. The research protocol was also listed on the Iranian Registry of Clinical Trials (IRCT) with code no. IRCT2020050904736N1.

### Participants

The statistical population included adult patients with opioid use disorder undergoing MMT. The inclusion criteria in this study were the willingness to participate, age over 18, literacy, drug use confirmed by positive urine tests, taking methadone as supervised by a physician for at least three months, and no chronic mental illnesses, such as psychosis, bipolar disorder, and schizophrenia. As well the exclusion criteria were the participants’ decline to continue the study for any reason, changes to the treatment plan, and the use of zinc-containing compounds prior to the intervention program.

**Fig. 1:**
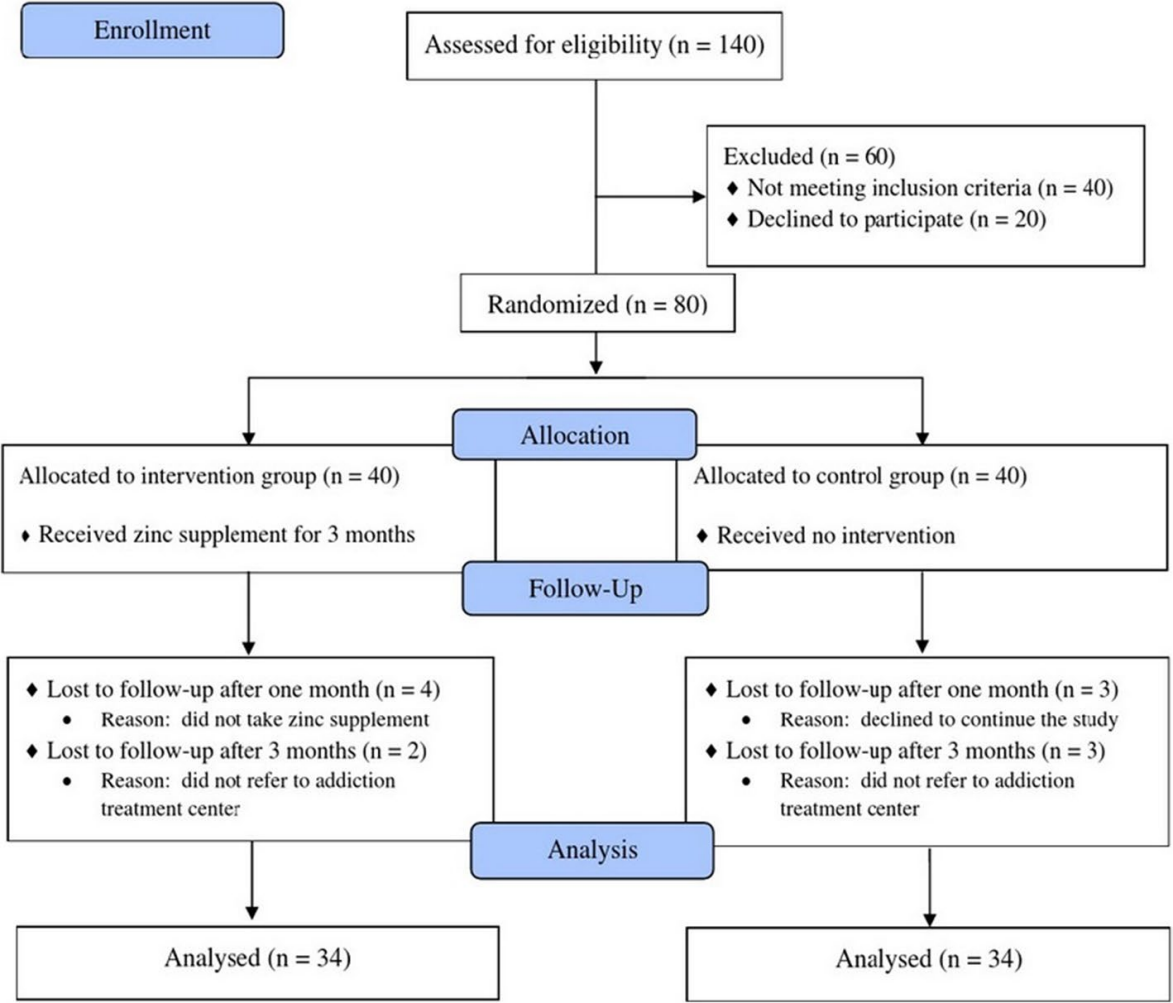
The research process according to the CONSORT flow diagram (2010)

The sample size was thus estimated at 32 individuals in each group, with reference to a similar study with 80% test power and a 95% confidence interval. Considering the 20% sample loss, the final sample size in each group was determined to be 40. The researcher then referred to the selected addiction treatment clinic for the sampling of the convenience type. For this purpose, first, the patients with opioid use disorder undergoing MMT were examined in terms of meeting the inclusion criteria. Next, the study objectives and phases, data confidentiality, and the voluntary basis of entering into and withdrawing from the research process were presented by holding face-to-face meetings with the eligible individuals. Afterward, those who agreed to participate in the study signed an informed consent form. Finally, out of 140 patients with opioid use disorder referred to the addiction treatment clinic concerned, 100 individuals with a history of at least three months of receiving methadone met the inclusion criteria, of which 80 ones agreed to participate in the study. A randomized block design was further utilized to allocate the samples to the intervention and control groups. The random allocation sequence (with 20 blocks of four) was accordingly established using the Random Allocation Software. For the act of concealment, some closed envelopes containing cards with the letters A (for the intervention group) and B (for the control group) were prepared according to an encoded allocation sequence. An envelope was then opened by an outsider who had no role in the study and did not know about the card inside for each individual. Based on the card inside, the patients with opioid use disorder were allocated to one of the intervention or control groups. Of note, only the data analyst was blinded to the allocation of the participants to the groups in this study to ensure that all the analytical decisions were made without bias. The CONSORT flow diagram of the study is illustrated in Fig. 1.

### Data collection

The study data were collected using the Demographic Survey Form (DSF), the Relapse Prediction Scale (RPS), and the Depression, Anxiety, and Stress Scale 21 (DASS-21) before, one month after, and three months after the intervention (i.e., the completion of the intervention program).

### DSF

The DSF consisted of items focused on the participants’ age, gender, marital status, living conditions (single or with family), smoking status (cigarettes or hookahs), education level, and occupation.

### RPS

The 45-item RPS was administered to assess the PoR. Each item included a situation in which the respondent could rate the likelihood of drug use and drug craving [19], on a five-point Likert-type scale (including 0 = none, 1 = weak, 2 = moderate, 3 = strong, 4 = very strong), with the minimum and maximum scores of zero and 180, respectively. Thus, the score between 0 and 60 indicated a low relapse rate, values from 60 to 90 predicted a moderate relapse rate, and a score above 90 implied a strong relapse rate. Previous studies further revealed that the internal consistency of the Persian version of this scale was between 0.74 and 0.91 [20–22].

### DASS-21

The DASS-21 was implemented to measure the MH status of the study participants. Lovibond and Lovibond [23] initially developed this 42-item scale, and its short form contained 21 items to assess depression, anxiety, and stress (viz. seven items in each case), using a four-point Likert-type scale (including 0 = never, 1 = sometimes, 2 = often, 3 = always). The total score of the questionnaire was calculated by the sum of the scores of the seven relevant items and then multiplied by two [24]. The psychometric properties of this scale had been examined in previous studies, and its internal consistency had been reported using Cronbach’s alpha coefficient of 0.74–0.90 and the two-week test–retest reliability of 0.74 [25]. For the Persian version of this scale, the mean Cronbach’s alpha coefficient has been 0.96 for the subscales, and the two-week test–retest reliability of 0.85 has been presented based on the Pearson correlation coefficient [26].

### Intervention program

The participants allocated to the intervention group were visited by a physician every two weeks, and then received a two-week supplement of zinc combined with methadone as planned. The daily dosage of the zinc supplement was about 12 mg. The supplement used in this study was Suzin Zinc Sulfate, made by Alhawi Pharmaceutical Co., Iran (of note, each capsule of 110 mg of zinc sulfate was equivalent to 25 mg of zinc). To check the participants’ cooperation in the intervention group, as well as their regular zinc supplement intake, their families were contacted three times a week. The researcher also welcomed their questions and ambiguities.

On the other hand, the participants in the control group only received methadone, according to the treatment plan, and their visits were fixed every two weeks to check their methadone use by a physician or a nurse. Of note, no placebo was given to this group. All the study participants were also asked not to change their diet and type of medication at the end of the study. The methadone dose was registered in both intervention and control groups.

### Data analysis

The SPSS Statistics software package (ver. 23) was used for the data analysis. To this end, the data from the intervention and control groups were summarized descriptively, using frequency and percentage for the categorical variables and mean and standard deviation (SD) for the continuous ones. The Kolmogorov–Smirnov test was further implemented to examine the normal distribution of data. To evaluate the homogeneity of the study groups, independent-samples t-test, Chi-square test, and Fisher’s exact test were utilized. Moreover, repeated measures analysis of variance (ANOVA) was performed to compare the predictive scores of the PoR and MH in the study groups. All the inferential tests were completed at the significance level of 0.05.

## Results

In total, 80 individuals participated in the present study and were allocated to two groups, viz. intervention and control (40 people in each group). One month after the intervention program, four people in the intervention group were lost to follow-up due to no medication intake, and three participants in the control group were excluded as they declined to cooperate further. Moreover, two months after the onset of the study, two other participants in the intervention group and three people as the controls withdrew from the study since they did not attend the addiction treatment clinic. Finally, the data collected from 68 patients with opioid use disorder (that is, 34 individuals in each group) were analyzed (Fig. 1).

The mean ± SD age of the participants in the intervention group was 40.85 ± 8.31 years old, and that was 38.21 ± 9.21 in the controls. As well, 73.5% of the individuals in the intervention and control groups were male. Considering marital status, most participants in the intervention (70.6%) and control (64.7%) groups were married. Other demographic characteristics of the participants are provided in Table 1. In terms of the demographic variables, there was no statistically significant difference between both groups, so they were homogeneous.

**Table 1:** Demographic variables by study groups

**Variables**	**Subgroups**	**Intervention group** Frequency (percentage)	**Control group** Frequency (percentage)	***P*-value**
Marital status	Married	24 (70.6%)	22 (64.7%)	0.60^a^
	Single	10 (29.4%)	12 (35.3%)	
Tobacco use	Cigarette smoking	18 (52.9%)	17 (50.0%)	0.96^a^
	Hookah	10 (29.4%)	11 (32.4%)	
	None	6 (17.6%)	6 (17.6%)	
Occupation	Unemployed	6 (17.6%)	6 (17.6%)	0.16^b^
	Self-employed	2 (5.9%)	8 (23.5%)	
	Employee	6 (17.6%)	2 (5.9%)	
	Worker	9 (26.5%)	4 (11.8%)	
	Homekeeper	3 (8.8%)	4 (11.8%)	
	Retired	8 (23.5%)	10 (29.4%)	
Education Level	Less than diploma	24(70/6%)	22(64/7%)	.004^b^
	Diploma	4(11.8%)	12(35.3%)	
	University degree	6(17.6%)	0(0.0%)	

^a^Chi-square test, ^b^Fisher’s exact test

A comparison of the change trends in the RPS scores in the study groups is given in Table 2. Moreover, comparing the trend of the intragroup changes in respect of the likelihood of drug use, drug craving, and the RPS total score during the study in the control and intervention groups was not significant. However, comparing the intergroup change trends showed that the likelihood of drug use (*p* = 0.01), drug craving (*p* = 0.002), and the RPS total score (*p* = 0.002) in the intervention group was significant.

**Table 2:** A comparison of RPS scores in study groups

**Variables**	**Groups**	**Before intervention** Mean ± SD	**One month after intervention** Mean ± SD	**Three months after intervention** Mean ± SD	**P**^**1**^	P^3^	Partial eta square^a^
					**P**^**2**^		
likelihood of drug use	Control	73.11 ± 18.54	73.79 ± 17.26	77.97 ± 18.12	0.49	0.01	0.09
	Intervention	65.88 ± 21.26	64.94 ± 17.73	70.02 ± 23.47	0.35		
Drug craving	Control	75.61 ± 14.04	79.76 ± 12.48	76.94 ± 13.30	0.43	0.002	0.136
	Intervention	73.26 ± 15.18	69.323 ± 12.965	70.000 ± 14.034	0.38		
Total score	Control	148.73 ± 3.66	153.55 ± 20.81	154.91 ± 23.61	0.49	0.002	0.142
	Intervention	139.14 ± 29.99	134.26 ± 24.27	140.02 ± 27.98	0.41		

P^1^ = Significant for the trend mean values in the control group (Repeated measures ANOVA)

P^2^ = Significant for the trend mean values in the intervention group (Repeated measures ANOVA)

P^3^ = Significant for the total difference in means (before and after the intervention) between the intervention and control groups (Repeated measures ANOVA)

^a^Partial Eta Square interpret: 0.01 ∼ small, 0.06 ∼ medium, > 0.14 ∼ large

Furthermore, a comparison of the trend of changes in the DASS-21 scores in the study groups is presented in Table 3. In this sense, comparing the intragroup change trends in the intervention group demonstrated that depression (*p* = 0.01), anxiety (*p* < 0.001), stress (*p* = 0.001), and the DASS-21 total score (*p* = 0.001) were significantly descending during the study. However, in the control group, stress (*p* < 0.001) and the DASS-21 total score (*p* = 0.001) were increased. (The trend of the average score of stress and DASS-21 in the group control before and after the study increases). In addition, a comparison of the intergroup change trends showed no significant difference between both groups in respect of depression, anxiety, stress but the DASS-21 total score (*p* < 0.001) in the intervention group were significant than those in the control group.

**Table 3:** A comparison of DASS-21 scores in study groups

**Variables**	**Groups**	**Before intervention** Mean ± SD	**One month after intervention** Mean ± SD	**Three months after intervention** Mean ± SD	P^1^	P^3^	Partial eta square^a^
					P^2^		
Depression	Control	27.05 ± 7.97	27.29 ± 5.09	27.58 ± 5.34	0.89	0.87	0.013
	Intervention	29.47 ± 6.75	26.82 ± 6.15	26.29 ± 7.00	0.01		
Anxiety	Control	24.17 ± 7.09	25.58 ± 5.41	26.00 ± 6.51	0.139	0.39	0.021
	Intervention	26.64 ± 7.43	24.00 ± 6.47	21.38 ± 6.14	< 0.001		
Stress	Control	22.23 ± 7.13	25.29 ± 5.56	28.17 ± 6.57	< 0.001	0.08	0.044
	Intervention	24.67 ± 6.43	22.58 ± 6.77	20.64 ± 8.49	0.001		
Total score	Control	73.47 ± 12.25	78.17 ± 10.02	81.76 ± 13.08	0.001	< .001	0.390
	Intervention	80.79 ± 12.32	73.41 ± 13.61	68.32 ± 15.91	0.001		

P^1^ = Significant for the trend mean values in the control group (Repeated measures ANOVA)

P^2^ = Significant for the trend mean values in the intervention group (Repeated measures ANOVA)

P^3^ = Significant for the total difference in means (before and after the intervention) between the intervention and control groups (Repeated measures ANOVA)

^a^Partial Eta Square interpret: 0.01 ∼ small, 0.06 ∼ medium, > 0.14 ∼ large

## Discussion

This study aimed to investigate the effect of zinc supplementation on the PoR and MH in patients with opioid use disorder undergoing MMT, wherein the results indicated that zinc supplementation could reduce the PoR and improve the MH status in such individuals. As no studies had so far assessed the effect of zinc supplements on the PoR and MH among patients with opioid use disorder going through MMT, to the best of the authors’ knowledge, the results of the present study were reviewed based on the research outcomes in other populations and groups.

In this line, previous research had reported that the complications of opioid analgesics use, such as anorexia, constipation, gastroesophageal reflux disease, vomiting, and abdominal pain, could affect the gastrointestinal tract function and induce malnutrition as well as the deficiency of various minerals in the body, including zinc [27]. Based on the results of the present study, zinc supplement intake in the intervention group subsided drug craving, the likelihood of drug use and, in general, minimized the PoR in the patients with opioid use disorder experiencing MMT. To the best of the authors’ knowledge, there was no clinical study reflecting the effect of zinc supplementation on any PoR or other types of dependence. In a study on Morphine dependent rats, the authors suggested that oral zinc supplementation could lower addiction risk and zinc misbalances may reduce the craving for opioids by attenuating depression [15]. The results of the study could be thus somewhat related to the properties of zinc ions in diminishing the binding of opioids to receptors, as evidenced in laboratory studies [28, 29]. In 2021, Tantillo et al. indicated that the lack of zinc increases opioid consumption in patients undergoing total hip arthroplasty. As a result, zinc supplementation is regarded as a straightforward method for minimizing opioid use [30].

Moreover, zinc deficiency is able to have a significant effect on other mechanisms involved in relapses and even be associated with depression and anxiety [16], which may induce drug use [31].

The related literature also shows that a large number of patients with opioid use disorder subjected to MMT suffer from MH conditions, especially depression and anxiety [4, 5]. The results of the present study revealed that zinc supplementation improved MH (depression, anxiety, and stress) in patients with opioid use disorder undergoing MMT. The researchers, however, did not find any published studies investigating the effect of zinc supplement intake on the MH status of those receiving MMT. Therefore, such outcomes were compared with other populations. In line with the results of the present study, a systematic review and meta-analysis [32], along with a clinical trial [33], found that zinc supplementation had moderated the symptoms of depression in patients with opioid use disorder taking antidepressants. As well, in a cross-sectional study, an inverse relationship was observed between the recommended dietary allowance for zinc and mood swings, such as depression and anxiety in female students [16].

Even if the possible biological mechanisms that help improve MH (mainly the symptoms of depression and anxiety) via zinc supplementation are not yet fully understood, previous research has shown that zinc regulates neurotransmission, neurogenesis, the endocrine system, inflammation, and oxidative stress in signaling pathways is of utmost importance [34, 35]. The low levels of zinc in the body also disrupt both the production and function of neurotransmitters associated with impaired neurogenesis. Finally, once the important modulator of neurogenesis, viz. the brain-derived neurotrophic factor, is inhibited, neurotransmitters are disrupted; the grounds for MH disorders (i.e., depression) are provided [36]. Moreover, zinc in the hypothalamic–pituitary–adrenal axis plays an active role in regulating the body’s reaction to stress. The lower levels of zinc can thus stimulate this axis, elevating plasma cortisol and resulting in higher vulnerability to MH conditions [36, 37]. The literature also highlights the effect of zinc on such disorders through its involvement in the inflammatory process. Zinc deficiency accordingly interferes with antioxidant activity by reducing the production of superoxide dismutase and increasing the state of cellular oxidative stress, which in turn can augment inflammatory processes and inflammatory markers [38]. Increased proinflammatory cytokines may thus cause brain damage and then make changes in serotonin function in the brain, predisposing a person to depression [39].

### Limitations

To interpret the findings, there are some limitations in the present study that should be considered. First, this clinical trial was conducted in only one addiction treatment clinic located in an urban area of ​​Iran, which could reduce the generalizations to all patients with opioid use disorder undergoing MMT. Second, the PoR and MH here were assessed using self-report scales; however, the implementation of objective methods could be more beneficial in better assessing the impact of such intervention programs. Third, the effect of zinc supplementation in the present study on the PoR and MH status was not measured at long intervals after the completion of the intervention program (of note, the intervention lasted three months). Finally, the nature of the intervention in this study made it difficult to blind the participants.

### Implications for clinical practice

This study has some implications for clinical practice. In view of that, the findings highlight the effect of zinc supplementation on reducing the PoR and improving MH in patients with opioid use disorder experiencing MMT. Therefore, healthcare providers can introduce zinc supplements into the treatment plans for such individuals to help diminish their PoR and enhance their MH. However, further research is needed to replicate such outcomes. In addition, more studies are recommended to reflect on the challenges facing zinc administration in clinical practice from the perspective of healthcare providers and patients with opioid use disorder undergoing MMT, which can aid in making decisions for the inclusion of zinc supplements into the treatment plan of this group.

## Conclusion

In this study, zinc supplementation in patients with opioid use disorder subjected to MMT was investigated. Although the study results showed that zinc intake had the capability to positively influence relapse reduction and mental health in such people, more studies are needed before including this supplement in the treatment plans for this group.

## Data Availability

The data used in this study will be made available from the corresponding author upon reasonable request.

## Abbreviations

MMT: Methadone maintenance treatment
MH: Mental health
PoR: Probability of relapse
RPS: Relapse Prediction Scale
DASS-21: Depression, Anxiety and Stress Scale 21
DSF: Demographic Survey Form
SD: Standard deviation
ANOVA: Repeated measures analysis of variance
